# Pityriasis Rosea Following Pfizer-BioNTech Vaccination in an Adolescent Girl

**DOI:** 10.7759/cureus.27108

**Published:** 2022-07-21

**Authors:** Nouf F Bin Rubaian, Seereen R Almuhaidib, Shadan A Aljarri, Areen S Alamri

**Affiliations:** 1 Department of Dermatology, Imam Abdulrahman bin Faisal University, Al Khobar, SAU

**Keywords:** case report., pediatric, covid-19 vaccination, pfizer-biontech vaccination, pityriasis rosea

## Abstract

Pityriasis rosea (PR) is an acute self-limiting exanthematous skin disorder characterized by the presence of a primary solitary lesion called a herald patch and the subsequent development of diffuse papulosquamous lesions within 1 to 2 weeks. This is a case of COVID-19 vaccine-induced PR in the age group (12-18 years) that was recently approved for vaccination. We report a case of a 15-year-old otherwise healthy female with a history of 2 weeks of single oval primary plaque appearing on the right wrist 2 days after receiving the second dose of Pfizer-BioNTech vaccine, followed by diffuse and mild itchy skin eruptions spreading over the abdomen, back, chest, and extremities. The patient had no other symptoms and no PR risk factors. The patient was placed on 800 mg acyclovir five times a day and improved markedly after 1 week. As vaccine-induced PR/PR-like eruptions (PR-LE) is an uncommon phenomenon, we recommend further studies to determine the association between PR/PR-LE and COVID-19 vaccination.

## Introduction

Pityriasis rosea (PR) is the most common skin lesion in children and young adults [1]. PR has been estimated to account for 2% of dermatologic outpatient visits worldwide, with around 0.13% frequency in the United States [2]. PR is an acute, self-limiting, exanthematous disease of undetermined etiology [3]. Nonetheless, multiple studies have proved that viral agents, mainly human herpesviruses 6 and 7 (HHV-6/7), certain drugs, and rarely vaccines, may trigger PR [3]. PR typically begins with a primary solitary lesion called a “herald patch,” after which erythematous oval scaly eruptions occur, mainly on the trunk and extremities [1]. In most cases, PR lasts for approximately 2 to 8 weeks and resolves spontaneously [1]. The classical presentation of the disease can be diagnosed clinically without the need for biopsy confirmation [3]. Depending on the presentation, treatment options for PR patients can include topical steroids, oral antihistamine, acyclovir, erythromycin, and ultraviolet phototherapy [3].

In December 2019, a new highly infectious disease, later named coronavirus disease 2019 (COVID-19), emerged [4]. COVID-19 is a viral disease caused by severe acute respiratory syndrome coronavirus 2 (SARS-CoV-2) [4]. In the months following its discovery, COVID-19 spread rapidly, causing a global pandemic, and vaccines were developed to curb its spread [4]. Various cutaneous reactions to these vaccines have been observed, and an uncommon reaction reported is PR-like eruptions (PR-LE) [5].

To the best of our knowledge, we report the first case of PR following COVID-19 vaccination in the age group (12-18 years) recently approved for vaccination.

## Case presentation

**Figure 1:**
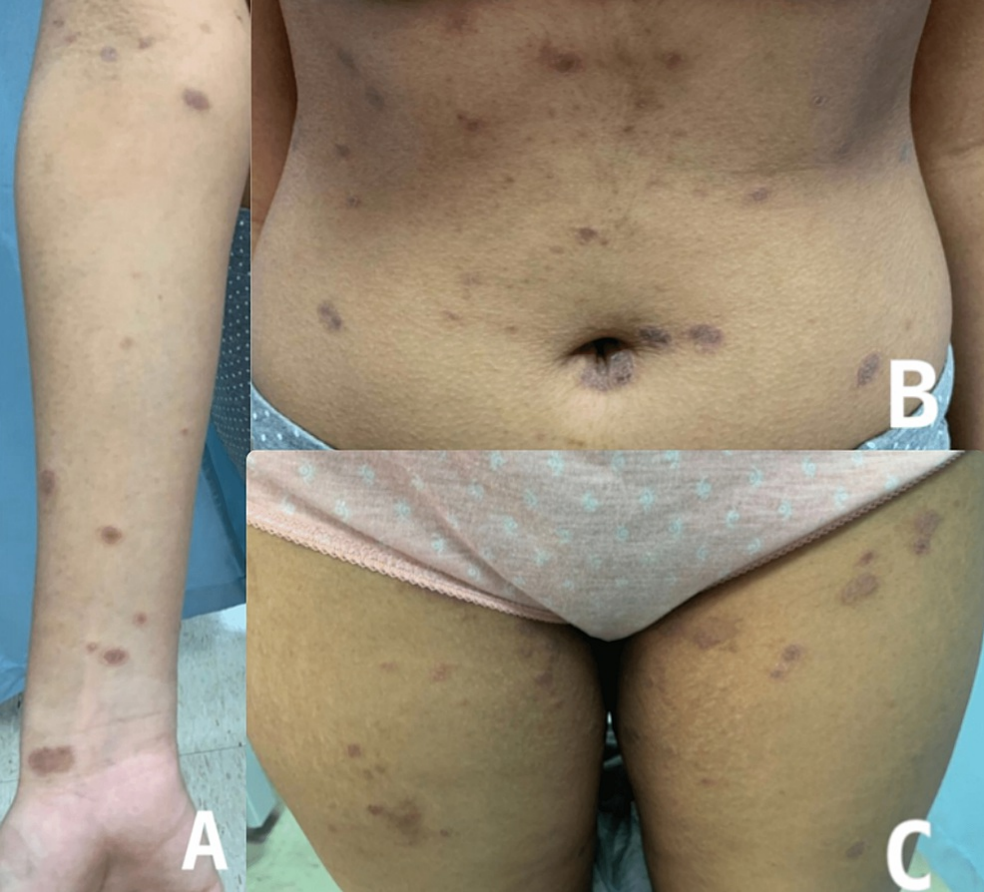
A: oval erythematous plaque with collarette peripheral scaling measuring 2 cm x 1 cm on the right wrist consistent with herald patch. Diffuse multiple oval to round erythematous scaly papules and plaques. A: right forearm, B: abdomen, C: thighs.

A 15-year-old otherwise healthy Saudi female presented to the dermatology clinic with a skin rash that appeared 2 days after her second dose of the Pfizer-BioNTech vaccine. It started with a single plaque on her wrist, followed by multiple papules and plaques on her chest, abdomen, back, upper limbs, and thighs (Figure 1). The skin rash was mildly itchy, with no accompanying fever or systemic symptoms. The patient denied any similar rash in her family. There was no history of contact with a COVID-19-infected patient and no history of medication used prior to the rash. The patient did not have symptoms of upper respiratory tract infection or any febrile illness preceding rash development. Dermatological examination showed an oval erythematous plaque with collarette peripheral scaling measuring 2 cm x 1 cm on the right wrist consistent with a herald patch. Also, there were multiple oval-to-round erythematous scaly papules and plaques on her chest, abdomen, back, arms, forearms, and thighs along Langer cleavage lines. The patient presented to our clinic one week after the rash appeared. She was very anxious about it, as she tried using topical fucicort cream prescribed by a private clinic with no improvement but rather an increase in skin eruptions. The diagnosis of PR secondary to the Pfizer-BioNTech vaccine was made based on the clinical assessment.

**Figure 2:**
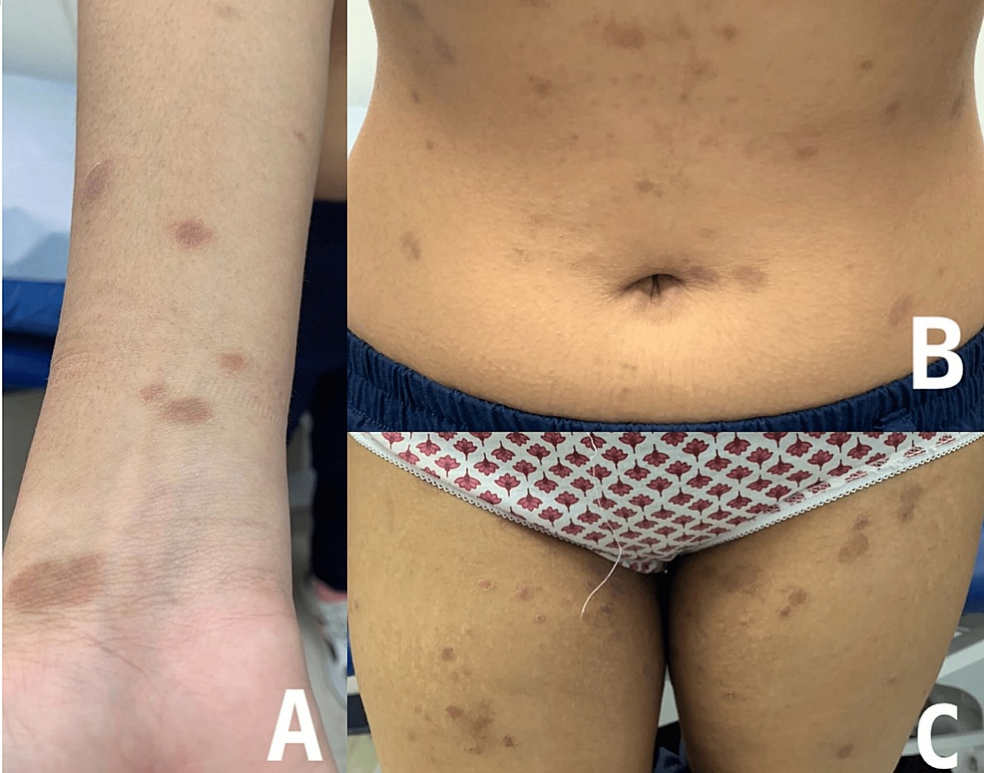
Lesions starting to improve after a 1-week course of acyclovir. A: right wrist, B: abdomen, C: thighs.

**Figure 3:**
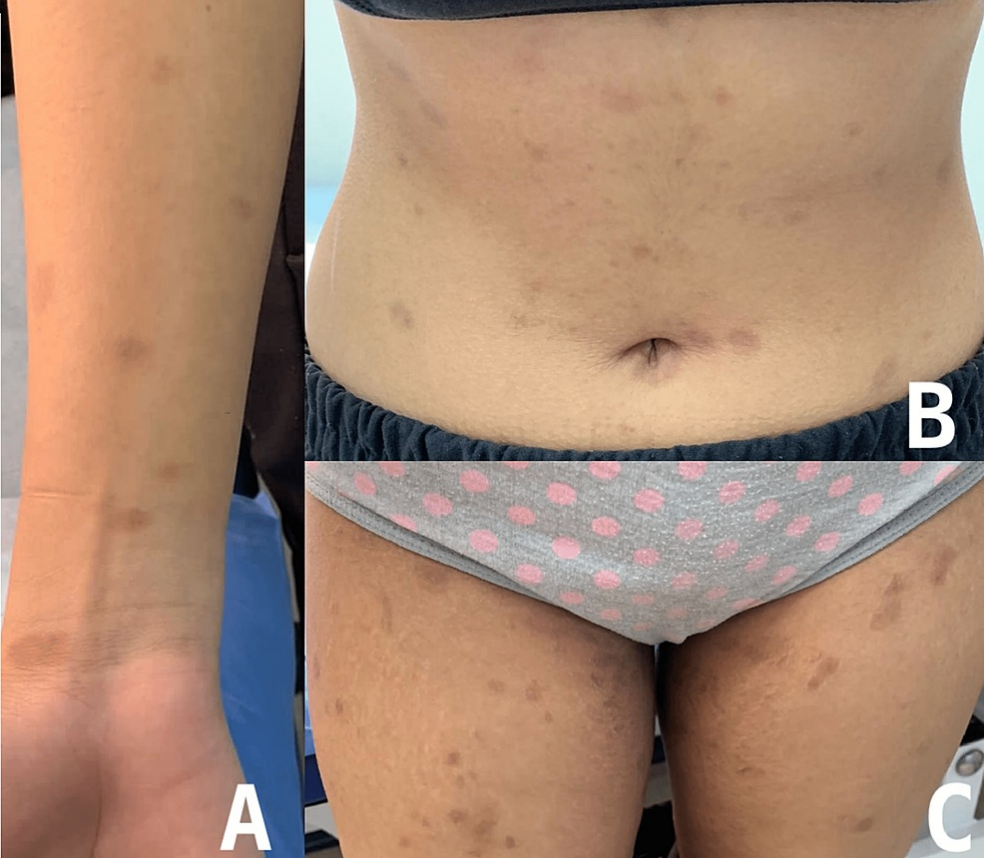
Post-inflammatory hyperpigmentation with complete resolution of pityriasis rosea rash. A: right wrist, B: abdomen, C: thighs.

The patient was treated with 800 mg of oral acyclovir five times a day for one week. Additionally, she was reassured that it was a benign rash that could persist for up to six to eight weeks and then spontaneously resolve. The patient was followed weekly for two weeks. In the first week of follow-up, the rash stopped appearing after completing the acyclovir course (Figure 2). In the second week of follow-up, the patient was left with post-inflammatory hyperpigmentation (Figure 3).

## Discussion

PR and PR-LE have been reported in the literature as uncommon cutaneous reactions to COVID-19 vaccinations [5]. Of all cutaneous reactions to COVID-19 vaccines, PR-LE was reported in 4.9% of cases [6]. PR is defined as an acute exanthematous skin disorder characterized by the presence of a herald patch and subsequent development of oval papulosquamous lesions on the cleavage lines, also known as Langer’s lines, within one to two weeks [1]. In contrast, PR-LE can involve the mucous membranes, in which more pruritic diffuse and confluent lesions are identified, with no prior history of a herald patch or prodromal symptoms [7]. The development of PR and PR-LE occurs through the reactivation of HHV-6/7 and the administration of drugs, respectively [7]. Virological investigations for HHV-6/7 reactivation are negative in PR-LE, with the possible presence of blood eosinophilia and eosinophils in the histopathology of lesions [7]. Moreover, treatment options for PR patients start with reassurance, patient education, and pruritus symptomatic management by topical steroids or antihistamines. For severe cases, acyclovir or ultraviolet phototherapy can be used [3,8]. For our patient, we used acyclovir due to the patient's anxiety and the extent of the rash. Acyclovir can be started within two weeks of rash appearance. [8]

Previously reported cases of PR and PR-LE following COVID-19 vaccinations imply a causal link [9]. Vaccine-induced PR/PR-LE is not a recent event, as a few cases of smallpox, tetanus, poliomyelitis, influenza, papillomaviruses, diphtheria, hepatitis B, tuberculosis, pneumococcus, diphtheria-pertussis-tetanus, and yellow fever vaccinations have been described to cause PR/PR-LE eruptions [10]. The specific pathogenesis of vaccine-induced PR/PR-LE remains unknown, yet there are several hypotheses [10]. As vaccines are implicated in evoking a specific immune reaction to a certain infectious agent, the level of plasma cytokines increases, which may disturb the control of latent infections by T-cell-mediated immunity [10]. This, in turn, explains the possible reactivation of HHV-6/7 and the subsequent development of PR in the presence of vaccinations and, at present, COVID-19 vaccinations. PR-LE related to vaccines may be associated with delayed hypersensitivity reactions, similarly seen with the use of drugs [10]. Molecular mimicry with a viral epitope is an additional suggested mechanism that leads to a skin reaction mediated by T-cell immunity [10].

Several types of COVID-19 vaccinations have been reported to cause PR/PR-LE. Busto-Leis et al. reported the first two cases of PR following the administration of the Pfizer-BioNTech COVID-19 vaccine [11]. The Oxford-AstraZeneca vaccine, also known as Covishield, was implicated in two cases of PR in which the eruption developed within four days following the administration of the first dose [3,9]. PR was also seen after the use of the CoronaVac COVID-19 vaccine, an inactivated SARS-CoV-2 vaccine [4]. Thirty-one cases of PR were reported in the first case series regarding the eruption of PR following COVID-19 vaccination by Temiz et al. [12]. The Pfizer-BioNTech messenger RNA (mRNA) vaccine and CoronaVac vaccine accounted for 45.2% and 54.8%, respectively [12]. The majority of cases had an average onset of post-vaccination PR in the 13 days following their first dose of the vaccine [12]. In a cross-sectional study conducted by Català et al. regarding SARS-COV-2 vaccine-induced cutaneous manifestations, PR-LE accounted for 4.9% [6]. Of these, 55% were due to Pfizer-BioNTech, followed by Moderna (25%) and AstraZeneca (20%) vaccines [6]. Sixty percent of the cases experienced the eruption of PR-LE after their first dose within an average onset of six days [6]. In our case, the patient had a PR eruption within two days of the administration of the second dose of the Pfizer-BioNTech vaccine.

The diagnosis of PR is mainly based on history and physical examination [3]. Our case was diagnosed clinically as typical PR. The patient developed a herald patch on her right wrist, followed by multiple papulosquamous lesions distributed on her arms, forearms, chest, back, abdomen, and thighs.

PR has been described in the literature as a self-limiting dermatosis [3]. Symptomatic treatment for pruritis could be administered, including emollients, antihistamines, and topical steroids [1]. As far as we know, none of the reported cases of COVID-19-induced PR/PR-LE have used an antiviral medication. In our case, acyclovir was used in the treatment of PR, and the lesions resolved within one week of treatment.

## Conclusions

We reviewed the literature regarding similar cases of PR/PR-LE following COVID-19 vaccines and reported a case of COVID-19 vaccine-induced PR in the age group (12-18 years) recently approved for vaccination. Herein, Pfizer-BioNTech-induced PR in a 15-year-old healthy female was treated with acyclovir. Because vaccine-induced PR/PR-like eruptions are an uncommon entity, we recommend further studies to determine the association between PR/PR-LE and COVID-19 vaccination.
